# Host Induced Gene Silencing Targeting *Aspergillus flavus aflM* Reduced Aflatoxin Contamination in Transgenic Maize Under Field Conditions

**DOI:** 10.3389/fmicb.2020.00754

**Published:** 2020-04-28

**Authors:** Yenjit Raruang, Olanike Omolehin, Dongfang Hu, Qijian Wei, Zhu-Qiang Han, Kanniah Rajasekaran, Jeffrey W. Cary, Kan Wang, Zhi-Yuan Chen

**Affiliations:** ^1^Department of Plant Pathology and Crop Physiology, Louisiana State University Agricultural Center, Baton Rouge, LA, United States; ^2^Food and Feed Safety Research Unit, United States Department of Agriculture – Agricultural Research Service, Southern Regional Research Center, New Orleans, LA, United States; ^3^Cash Crops Research Institute, Guangxi Academy of Agricultural Sciences, Nanning, China; ^4^Department of Agronomy, Iowa State University, Ames, IA, United States

**Keywords:** *Aspergillus flavus*, RNAi, host induced gene silencing, *aflM*, aflatoxin, transgenic, maize, droplet digital PCR

## Abstract

Maize (*Zea mays* L.) is one of the major crops susceptible to *Aspergillus flavus* infection and subsequent contamination with aflatoxins, the most potent naturally produced carcinogenic secondary metabolites. This pathogen can pose serious health concerns and cause severe economic losses due to the Food and Drug Administration (FDA) regulations on permissible levels of aflatoxins in food and feed. Although biocontrol has yielded some successes in managing aflatoxin contamination, enhancing crop resistance is still the preferred choice of management for long-term sustainability. Hence, host induced gene silencing (HIGS) strategy was explored in this study. The *A. flavus* gene *aflM* encoding versicolorin dehydrogenase, a key enzyme involved in the aflatoxin biosynthetic pathway, was selected as a possible target for suppression through HIGS. An RNAi vector containing a portion of the *aflM* gene was constructed and introduced into immature B104 maize zygotic embryos through *Agrobacterium* transformation. PCR analysis of the genomic DNA from T0 leaf tissue confirmed the presence of the transgene in six out of the seven events. The seeds from the lines that showed reduced aflatoxin production in laboratory aflatoxin kernel screening assay (KSA) have been increased from T1 to T4 generation in the past four years. Changes in aflatoxin resistance in these transgenic kernels have been evaluated under both field and laboratory conditions. The T2 generation kernels containing the transgene from two events out of four examined had less aflatoxin (*P* ≤ 0.01 and *P* ≤ 0.08) than those without the transgene. Field-inoculated homozygous T3 and T4 transgenic kernels also revealed lower levels of aflatoxins (*P* ≤ 0.04) than kernels from the null (segregated non-transgenic samples) or B104 controls. A similar result was observed when the harvested T3 and T4 homozygous transgenic kernels were evaluated under KSA conditions without inoculation (*P* ≤ 0.003–0.05). These two events were crossed with LH195, LH197, LH210, and PHW79 elite breeding lines and the resulting crosses supported less aflatoxin (*P* ≤ 0.02) than the crosses made with non-transgenic lines. In addition, significantly higher levels of *aflM* gene-specific small RNAs were detected in the transgenic leaf and kernel tissues, indicating that the enhanced aflatoxin resistance in the homozygous transgenic kernels is likely due to suppression of *aflM* expression through HIGS.

## Introduction

Maize (*Zea mays L*.) is one of the major agricultural crops grown worldwide on about 191.2 million ha of land in 2018 with the United States accounting for 17.3%. Global maize production reached 1,078 million metric tons (MMT) in 2017 and was expected to reach 1,123 MMT in 2018, with United States maize production accounting for 32.6–34.4%, according to the latest released report release by USDA-Foreign Agricultural Service^1^. However, global maize production is under constant threat of various diseases. One of them is infection by *Aspergillus flavus* and subsequent contamination with aflatoxins, the most potent naturally occurring toxic secondary metabolites, which are known to cause liver cancer in humans (Squire, 1981; Robens and Richard, 1992; IARC, 2012; Moradi et al., 2015).

Aflatoxin contamination has led to public outbreaks of aflatoxicosis. In 2004, hundreds of people died from consuming aflatoxin contaminated maize in Kenya and hundreds of dogs in the United States died in 2006 from eating aflatoxin contaminated feed (Richard, 2008). Currently there are no effective controls that can completely eliminate aflatoxin contamination in maize and other susceptible crops. The use of chemicals to control *A. flavus* infection and subsequent aflatoxin contamination is ineffective (Wheeler et al., 1991; Bruns and Abbas, 2006). Biocontrol is the only measure known to reduce aflatoxin contamination in the field, but its efficacy varies depending on moisture and timing of application (Moore et al., 2011). Although conventional breeding has greatly improved yields, elite breeding lines remain susceptible to aflatoxin contamination. Transferring polygenic resistance currently available in maize into elite breeding lines has been met with limited success due to linkage drag and incomplete resistance (Warburton et al., 2011; Mylroie et al., 2013).

Several studies have found that small RNAs, including both small interfering RNA (siRNA) and micro RNA (miRNA), travel between cells via plasmodesmata and systemically throughout the plant as mobile silencing signals, to regulate cellular processes, host defense, transcription and translation (Dunoyer et al., 2010; Pyott and Molnar, 2015; Rosas-Diaz et al., 2018). Several studies have also found that siRNA in the diet or medium can be transported across cellular membranes and affect target gene expression in *Caenorhabditis elegans* (Tabara et al., 1998) or *Aspergillus nidulans* (Khatri and Rajam, 2007), respectively. Further studies demonstrated movement of siRNA molecules between a parasite and its host plant (Tomilov et al., 2008), or between herbivorous insects and the host plant engineered to express dsRNAs targeting vital insect genes (Baum et al., 2007).

The major breakthrough in applying RNAi to control plant fungal diseases came from two studies. Tinoco et al. (2010) reported the suppression of *gus* gene expression in a GUS-transformed fungus *Fusarium verticillioides* when it was inoculated onto transgenic tobacco plants expressing an RNAi construct targeting the *gus* gene. Another study was by Nowara et al. (2010) who reported reduced infection by the powdery mildew fungus *Blumeria graminis* by expressing a silencing construct targeting the fungal effector gene *Avra10* in susceptible barley and wheat. This cross-kingdom RNAi based gene silencing phenomenon is called host induced gene silencing (HIGS), which has been demonstrated to successfully suppress disease development caused by fungi (including biotrophs, hemibiotrophs, and necrotrophs) as well as oomycetes (Govindarajulu et al., 2015; Park et al., 2016; Song and Thomma, 2016). Panwar et al. (2013) reported suppression of the wheat leaf rust fungus, *Puccinia triticina* when genes involved in pathogenicity were targeted. Later, Ghag et al. (2014) showed that transgenic banana producing siRNAs targeting vital fungal genes increased its resistance against *Fusarium oxysporum* f.sp. *cubense*. Jahan et al. (2015) further demonstrated successful control of *Phytophthora infestans* in potato using the same strategy. This strategy also improved plant resistance in a recent study against verticillium wilt, an economically important and notoriously hard to control disease that affects a wide range of host plants (Song and Thomma, 2016). These studies convincingly demonstrated that small RNA trafficking between plants and fungal pathogens provides a new and powerful tool to control plant diseases. In addition, some limited success of using HIGS to suppress aflatoxin production in maize by targeting *aflR* (encoding a key regulator of aflatoxin biosynthetic pathway) or *aflC* (encoding a polyketide synthase involved in the initial steps of aflatoxin biosynthesis) or *amy1* (encoding an alpha-amylase involved in fungal infection) of *A. flavus* has been reported (Masanga et al., 2015; Thakare et al., 2017; Gilbert et al., 2018). The lack of field confirmation and/or possible off-target effects of these studies, however, weakened the validity of these RNAi-based gene silencing strategies in managing aflatoxin contamination in maize.

Therefore, the objectives of the present study are to suppress through HIGS a different aflatoxin biosynthetic pathway gene *ver-1* (*aflM*), which was highly expressed and was involved in the later steps of aflatoxin biosynthesis (Yu, 2012), to (a) determine its ability in reducing aflatoxin contamination in progenies under both laboratory and field conditions, (b) determine whether the transgene can reduce aflatoxin production when transferred to elite inbred lines, and (c) determine whether the reduced aflatoxin contamination was due to the presence of gene specific small RNA from the HIGS construct.

## Materials and Methods

### Construction of HIGS Vector for Suppressing *aflM* Gene Expression

*AflM* (*ver1*) from *A. flavus* AF13 (gene accession number XM_002379900) was selected in this work. This gene encodes a versicolorin dehydrogenase that is involved in the conversion of versicolorin to demethyl-sterigmatocystin in later steps of aflatoxin biosynthesis (Yu et al., 2004; Yu and Ehrlich, 2011). To clone the gene into a Gateway-based vector (Chen et al., 2010), the 5′ and 3′ arms were selected from the coding region of the versicolorin dehydrogenase gene and were amplified using PCR with homologous recombination sites (italicized) attached to the end of the gene-specific primers (Supplementary Table 1). Briefly, the 5′ arm was amplified with attB4-Ver1F and attB1-Ver1R using the *A. flavus ver1* cDNA clone as a template, and the 3′ arm was amplified with attB2-Ver1F and attB3-Ver1R in a similar manner. The 5′ and 3′ arms were then ligated into pDONR P4-P1R and pDONR P2R-P3 (Invitrogen, Carlsbad, CA, United States), respectively, through BP clonase reactions, according to the manufacturer’s instruction. The resulting vectors were named pENTR-L4-5′arm-R1 and pENTR-R2-3′armL3, respectively. A MultiSite Gateway LR recombination reaction was performed with the four vectors pBS-d35S-attR4-attR3, pENTRL4-5′arm-R1, pDONR221-PR 10-intron-CmR (Chen et al., 2010), and pENTR-R2- 3′arm-L3, according to the manufacturer’s instructions. The reaction mixture was transformed into TOP10 *Escherichia coli* cells and selected on LB plates containing 100 mg/mL ampicillin and 30 mg/mL chloramphenicol. The resulting vector pBS-aflM-RNAi (pBS-d35S-attB4-5′arm-attB1-PR 10 intronCmR-attB2-3′arm-attB3) was then verified through restriction digestion and sequencing before digesting the vector with *Eco*RI and *Sac*I to remove the DNA region containing the aflM-RNAi cassette, which was then ligated into the corresponding sites of pTF102 (Frame et al., 2002), to generate the final RNAi vector pTF102-aflM-RNAi, which was further verified through digestion, before being used in maize transformation.

### Transformation of HIGS Vector Into Maize

Genetic transformation of maize inbred B104 was performed by plant transformation facility (PTF) of Iowa State University as described by Frame et al. (2000). Regenerable type I calli were subcultured, and fertile transgenic plants were recovered following selection on bialaphos-containing medium (Frame et al., 2000; Chen et al., 2010). The regenerated transgenic plants were pollinated with pollen from B104 between April and May of 2013, and ears from all seven independent transgenic events were harvested in June 2013.

### Confirmation of Transformation and Target Gene Expression

Genomic DNA was isolated from ground leaf tissues (100 mg) developed from transgenic cali or kernels of all seven independent transformation events using a modified CTAB method as describe by Doyle and Doyle (1987). The quality and quantity of the isolated total DNA was determined using a Nano-Drop ND-1000 Spectrophotometer (Thermo Fisher Scientific, Wilmington, DE, United States). DNA was diluted to the same concentration (50 ng/μL) and used as a template for PCR using specific primers corresponding to the *aflM* gene (Ver-1-F and Ver-1-R, Supplementary Table 1). The reaction was prepared at 1× final concentration in a 20 μL volume containing 0.4 μM of each primer and 1 μL of template. Expression of the target gene in developing leaves of young transgenic maize plants was confirmed using reverse transcriptase polymerase chain reaction (RT-PCR). Total RNA was isolated from plants of all 7 events and B104 wild type, which was used as a negative control. A reverse transcription cDNA RT kit (Life Technologies, Carlsbad, CA, United States) was used for quantitative real-time polymerase chain reaction (qRT-PCR) analysis follow the manufacture’s protocol. cDNA was used as a template for real time PCR using RT-Ver-F and RT-Ver-R (Supplementary Table 1). The expression level of the maize 18S rRNA gene (accession AF168884) was used as an internal control to normalize the level of target gene expression. The amplification efficiency of each primer pair used in this study was determined through serial dilutions, and this was taken into account in calculating target gene expression if it was outside the ideal efficiency range. The transgenic events confirmed positive for transformation were used in the studies described below.

### Evaluation of Aflatoxin Resistance in Different Generations of Transgenic Maize Kernels

Of the seven dependent transformation events received from Iowa, three events with the highest (aflM10, aflM14, and aflM16) and one event with the lowest (aflM13) levels of *aflM* gene expression were selected for the initial screening of the T1 generation of transgenic maize kernels. After surface sterilization of the kernels as described in the kernel screening assay (KSA) by Brown et al. (1993), 10–15 kernels per event were inoculated with 4 × 10^6^ conidia/mL of *A. flavus* toxigenic strain AF13 (ATCC 96044, SRRC 1273), and incubated at 30°C under 100% humidity. After seven days of incubation, kernels were dried at 65°C for 72 h to stop the fungal growth and ground for aflatoxin extraction using MeOH as described by Sobolev and Dorner (2002). Aflatoxin was quantified using a high performance liquid chromatography (HPLC) according to Joshua (1993). Genomic DNA was also isolated from ground powder of individual kernels after aflatoxin extraction to determine whether it contained the target gene or not.

Another fifteen T1 kernels from each of the above four events were sown in pots filled with potting mix (Marysville, OH, United States) in a greenhouse for seed increase in spring of 2015. Five to eleven seedlings from each event that were verified by PCR to contain the *aflM* gene were transplanted to a field for self-pollination by hand. Twenty-five kernels per event from the resulting T2 ears were tested for aflatoxin accumulation using KSA as described above. DNA isolation and verification for presence of the transgene was conducted on individual kernels for which aflatoxin data were obtained. T2 seeds (45 kernels/event) were increased to T3 in the field in spring of 2016, and from T3 to T4 in the field (60 kernels/event) in 2017 for two of the events (aflM14 and aflM16). In 2018, homozygous lines of these two events were crossed with four elite inbred lines (LH195, LH197, LH210, and PHW79) to determine whether the transgene can reduce aflatoxin production in the resulting crosses.

### Transgene Copy Number Assessment Using Real Time PCR and Droplet Digital PCR

Besides PCR confirmation of the presence of the target gene in genomic DNA extracted from transgenic seedling leaf tissues, transgene copy number (C) as described below was also determined for T0 leaf tissues collected from each transformation events that contained the target gene determined using real time PCR. TaqMan real-time PCR primers (Supplementary Table 1) specific to the *aflM* and to the endogenous single copy alcohol dehydrogenase gene (*adh1*) as a reference were used to quantify the relative ratios of *aflM*/*adh1* with the fluorogenic TaqMan probes. Real-time PCR was performed in an ABI Prism 7000 Sequence Detection System (Applied Biosystems, Foster City, CA, United States) in a final volume of 25 μL containing 1 × TaqMan Universal PCR Master Mix (Applied Biosystems), 200 nM of each primer, 100 nM of probe and 150 ng of genomic DNA under the following conditions: 50°C for 2 min, 95°C for 10 min, and 40 amplification cycles of 95°C for 15 s, and 55–60°C for 1 min depending on primers. Three technical replicates were included for each sample. Copy number was calculated as C = 2^*T*0^ *^*Ct*^*^(^*^*adh*1**^*^)–T0^ *^*Ct*^*^(^*^*aflM*^*^)^. Here, T0 *Ct* (*adh1*) is the threshold cycle number of the *adh1* reference gene in T0 leaf tissue. To distinguish between heterozygous and homozygous plants among the T2 seedlings, Zygosity (*Z*) was calculated by comparing the *Ct* values of T2 plants to T0 plants from the same events using the following equation: *Z* = 2^[T2^ *^*Ct*^*^(^*^*adh*1**^*^)^^–^^*T*2^ *^*Ct*^* ^(^*^*aflM*^*^)]^^–^^[*T*0^ *^*Ct*^*^(^*^*adh*1**^*^)^^– T0^ *^*Ct*^*^(^*^*aflM*^*^)]^ (Bubner and Baldwin, 2004).

To obtain more precise assessment of transgene copy number, the droplet digital PCR was also performed on the genomic DNA extracted from following samples: aflM14 (T0), aflM14 (T4), aflM16 (T0), aflM16 (T4), and aflM17 (T0) using the same real-time PCR primer and probe sets with the *bar* gene as the target gene and the *adh1* gene as a reference to quantify the transgene copy number in the T0 and T4 (homozygous) transgenic plants at the Interdisciplinary Center for Biotechnology Research, University of Florida, Gainesville, FL (Hindson et al., 2011; Głowacka et al., 2016; Xu et al., 2016; Collier et al., 2017).

### Transfer of the Transgene Into Elite Inbred Lines for Field Evaluation

To further verify whether the reduced aflatoxin production observed in the homozygous transgenic lines was due to the presence of the transgene, two non-stiff stock (LH210 and PHW79) and two stiff-stock (LH195 and LH197) elite inbred lines were pollinated with pollen from T4 generation homozygous and null aflM14 and aflM16 plants, and the resulting ears were inoculated 2 weeks after pollination at the Louisiana State University Agricultural Center Botanic Gardens, Baton Rouge, LA.

For field evaluation of the self-pollinated T3 and T4 generation of aflM14 and aflM16 events, homozygous, heterozygous, and non-transgenic plants grown in 2016 and 2017, respectively, and of crosses of the above four elite lines with homozygous plants and null of aflM14 and aflM16 in 2018, 8–10 ears from each line were inoculated with 3.4 mL per ear of *A. flavus* AF13 conidial suspension at four injection sites in the mid-ear using an Indico tree-marking gun (Forestry Suppliers, Jackson, MS, United States) with a 15-gauge hypodermic needle. Kernels from non-inoculated ears in the field were also collected each year and used as controls. The inoculum concentration used for field inoculation in 2016 was 4 × 10^6^ conidia/mL in 0.01% (w/v) SDS, which was adjusted to 1 × 10^5^ conidia/mL for 2017 and 2018 due to extremely high levels of aflatoxins detected in inoculated kernels from 2016. Four intact kernels surrounding the inoculation sites were recovered after maturing and used for aflatoxin extraction and analysis for 2016 and 2017. For crosses in 2018, at least eight ears per treatment were collected. Kernels from half of each ear were mixed and ground, and three subsamples were analyzed for aflatoxin levels using HPLC.

### Aflatoxin Extraction and Quantification Using HPLC

When multiple maize kernels were used for aflatoxin analysis, they were first ground into a fine powder with a coffee mill (Mr. Coffee) and then ∼60 to ∼1000 mg of ground powder was weighed and added to a 50 mL flask containing 25 mL of an 80: 20 methanol: water (HPLC grade) mixture, which was shaken at approximately 112 rpm at room temperature for 1 h. The extract was then filtered through 100-mm No. 1 Whatman filter paper into a 50-mL glass beaker. One hundred microliter of the extract was then diluted 10 fold with 100% methanol in a 1.5 mL tube and mixed well before being filtered through a 1.5-mL alumina-basic column (Sobolev and Dorner, 2002) and used for injection into HPLC for aflatoxin analysis.

The aflatoxin was quantified by reversed-phase HPLC as described in Sweany et al. (2011). Ten microliters of each sample was separated using a Waters e2695 HPLC (Waters Corp., Milford, MA, United States) with a Nova-Pak C18 4 μm 3.9 × 150 mm column at 38°C. The mobile phase was methanol: water (37.5: 62.5) at a 0.8 mL/min flow rate. Each sample was run for 16 min with the aflatoxin B_1_ (AFB_1_) peak emerging at approximately 13.5 min. The detection and quantification of aflatoxin was achieved through an in-line post-column derivatization using a UV right in a Photochemical Reactor for Enhanced Detection (Aura Industries Inc., New York, United States) followed by excitation at 365 nm wavelength and 440 nm emission with a Waters 2475 FLR Detector (Waters Corp., Milford, MA, United States) (Joshua, 1993). Empower software (Waters Corp., Milford, MA, United States) was used to calculate the area under the AFB_1_ peak. The peaks were manually assigned and aflatoxin quantity was calculated based on a calibration curve calculated from 4 replications of serial diluted AFB_1_ standards at 1, 5, 50, 500, and 1000 ng/mL. The average of AFB_1_ from the three injections of each dilution was used for standard curve calculation.

## Small RNA Library Construction, Sequencing and Bioinformatics for Detecting Gene Specific Small Rna

Total RNAs were isolated from T0 leaf tissues collected in 2013 of aflM14, aflM16, and aflM11 (null), T3 leaf tissues of aflM14H, aflM16H, and B104, and the immature maize kernels of the T4 plants collected from homozygous and null of aflM14 and aflM16 as well as B104 14 days after self-pollination. After grinding into powder, maize kernel RNA was extracted by TRIzol reagent according to the manufacturer’s instructions and then cleaned with RNAeasy Plant Mini Kit (QIAGEN, Hilden, Germany). The total RNA from maize leaf tissues was isolated by RNeasy Plant Mini Kit (QIAGEN, Hilden, Germany). The total RNA was checked for quality using a Nanodrop for small RNA library construction. Indexed sRNA libraries were constructed from the enriched sRNA fractions with the TruSeq Small RNA Library Preparation Kits (RS-200-0012, Illumina, San Diego, CA, United States) according to the manufacturer’s instructions. Indexed sRNA libraries were sequenced on the Illumina HiSeq 2500 platform at the Genomic Science Laboratory at North Carolina State University (Raleigh, NC, United States) in 2016 (T3 leaf tissues) and on Illumina HiSeq 4000 the Genomic Sequencing Core at UC Davis (Davis, CA, United States) in 2017 (T0 leaf tissues and T4 kernels), respectively. The adapters and indexes were trimmed using Cutadapt (Martin, 2011) version 1.12, and the reads were mapped to the maize and *A. flavus* genome sequences using Bowtie (Langmead et al., 2009; Langmead and Salzberg, 2012) to identify sRNAs with a perfect match. Awk command lines were used to extract small RNA specific to the targeted gene *aflM*. R (R Core Team, 2013) was used to generate a sRNA mapping figure.

### Statistical Analysis

Standard error was calculated using Excel (Microsoft Corp., Seattle, WA, United States). Statistical analysis was conducted using SAS version 9.4 (Statistical Analysis System, SAS Institute, Cary, NC, United States). Analyses of variance (ANOVAs) were calculated using Proc Mixed. *Post hoc* comparison of means was calculated using Turkey’s LSD means (Saxton, 1998). Significance in this study was defined by a confidence interval ≥95% (α = 0.05). Raw aflatoxin data were used directly in statistical analysis without transformation except those data from KSA of T1 and T2 generation and from PHW79 × aflM16H and PHW79 × aflM16N, which were log transformed to equalize variation between samples of the experiment.

## Results

### Construction and Transformation of HIGS Vector Into Maize

The HIGS vector was constructed as described in Supplementary Figure 1 and the final construct with inverted repeats of *aflM* fragment inserted was verified through digestions with *Eco*RV, *Mfe*I, and *Kpn*I restriction enzymes (Supplementary Figure 2). The fragment sizes estimated based on DNA markers were in agreement with the expected fragment sizes of the correctly assembled vector when it is digested with these enzymes, which were 2447 and 9085 bp; 299, 2447, and 8786 bp; and 1328 and 10204 bp, respectively. In addition, the correct assembly of the *aflM* inverted repeats in all four clones was also verified through sequencing with d35S-F, RNAi-R and PR10-F primers (Supplementary Table 1). This construct is capable of producing a 325-bp *aflM* dsRNA transcript with a 130-bp single-strand loop in the middle, once the transcript is processed in the host plant.

**FIGURE 1:**
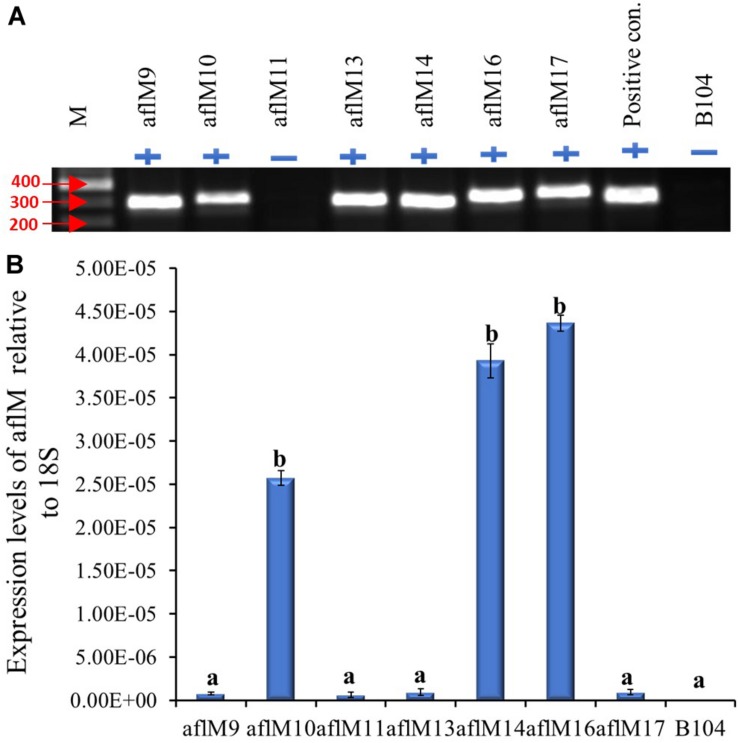
Determining the presence and level of expression of the target gene in the T0 transgenic leaf tissues. **(A)** PCR confirmation of the presence (+) or absence (−) of target gene in versicolorin (*ver-1, aflM*) RNAi vector transformed T0 leaf tissues. AflM RNAi plasmid DNA was used as a positive control and the genomic DNA from commercial maize line B104 was used as a negative (−) control. **(B)** Expression of transgene *aflM* in the T0 leaf tissue of various transformation events relative to 18S rRNA using real time PCR. AflM11 is negative for the transgene.

The construct was transformed into immature embryos of maize inbred line B104 through *Agrobacterium* infection in October of 2012. Twenty-three transgenic plants regenerated from seven independent transformation events were pollinated from April to May of 2013, and mature kernels were harvested in June 2013. Each event had one to six plants with a total of one to five ears per event. All events except aflM11 were confirmed positive for the presence of the target gene when genomic DNA from T0 plant leaf tissues was used as template (Figure 1A). Three of the positive transformation events (aflM16, followed by aflM14 and aflM10) showed significantly higher *aflM* target gene expression than the other events when the RNA extracted from T0 plant leaf tissues was examined using qRT-PCR (Figure 1B). AflM9, aflM13, and aflM17 had the lowest level of target gene expression (Figure 1B).

### Characterization of T1 and T2 Generations of Transgenic Seeds

**FIGURE 2:**
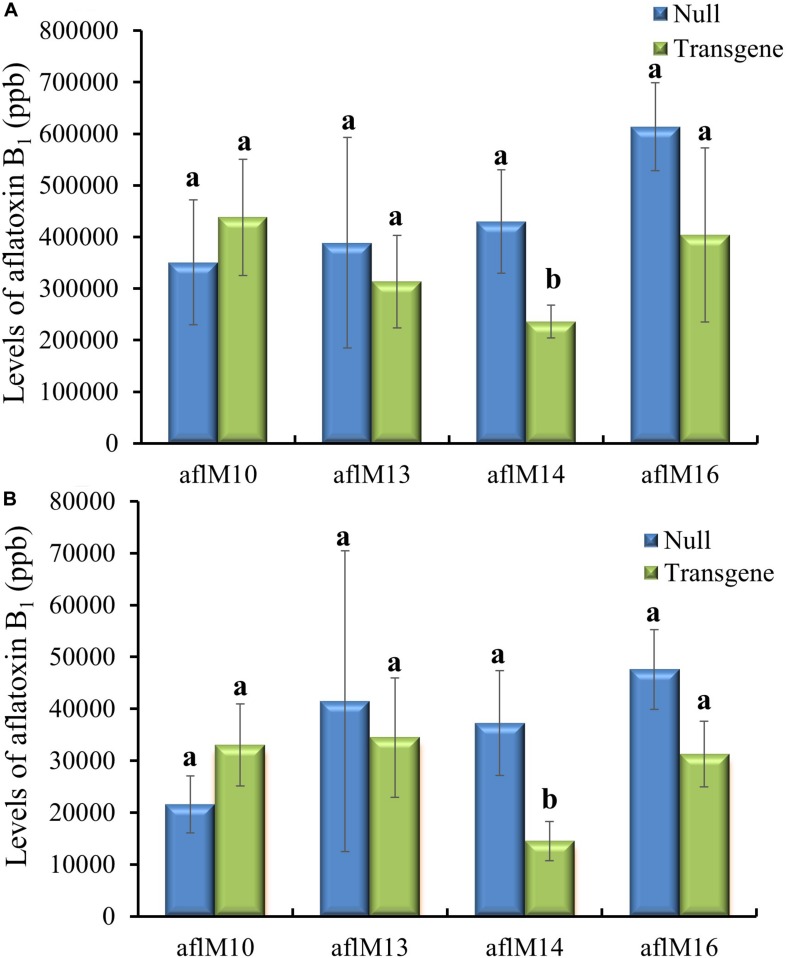
Aflatoxin production of the T1 **(A)** and T2 **(B)** generation of transgenic seeds containing *aflM* from four different events compared to null seeds under kernel screening assay (KSA) conditions. Data presented are the mean and standard errors of ten replicates for each event. Bars labeled with the same letters are not significantly different at *P* ≤ 0.05. Transgene represents the kernels that contain *aflM*. Null seeds for T1 are kernels from the same transformation events without the presence of *aflM*, and for T2 are segregating non-transgenic kernels from the same transformation events.

Twenty-one ears were produced from the six events that were confirmed positive for the transgene. The number of kernels ranged from 13 to 145 per ear, and average kernel weight ranged from 0.15 to 0.22 g (Supplementary Table 2). Ten to fifteen T1 generation transgenic kernels from each of the three events (aflM10, aflM14, and aflM16) with high levels of target gene expression and one event (aflM13) with very low level of target gene expression in the leaf tissue were selected for aflatoxin resistance analysis through KSA. Only the transgenic kernels from the aflM14 produced significantly less aflatoxin than the kernels without the transgene (null) (Figure 2A). T1 seeds from the aflM10, aflM13, aflM14, and aflM16 events were increased in the field in 2015 through self-pollination to the T2 generation for further analysis. Up to 60% less aflatoxin B_1_ production was observed in kernels from the T2 generation of aflM14 compared with the null (segregating non-transgenic) when analyzed using KSA (Figure 2B). The kernels from the T2 generation of aflM16 also produced less aflatoxin (*P* = 0.08) than the kernels from the null control (Figure 2B).

### Phenotypic Assessment of Transgenic Plants

**FIGURE 3:**
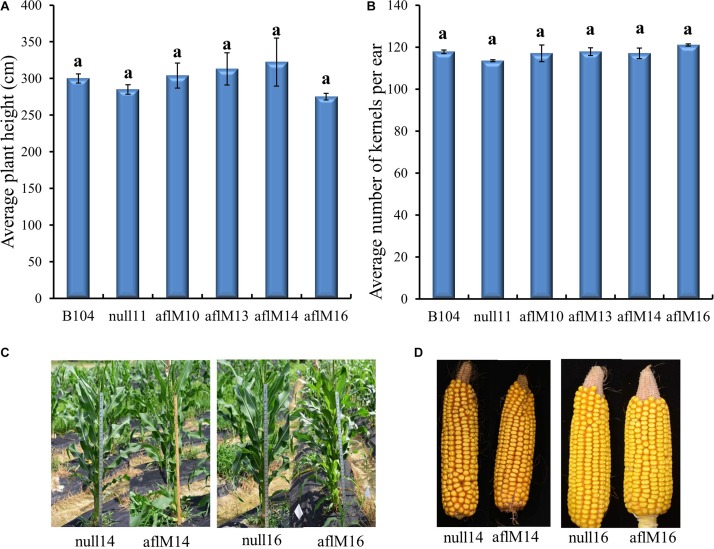
Phenotypic assessment of transgenic plants and mature ears. **(A)** Average plant height in aflM10, aflM13, aflM14, and aflM16 in comparison to the null11 and B104 (wild type) controls at 50 days after planting at T1 generation. **(B)** Average number of T2 generation kernels per ear of aflM10, aflM13, aflM14, and aflM16 compared to null11 and B104 (wild type). Vertical bars represent standard errors of the means. Means with the same letters are not significantly different between treatments at *P* ≤ *0.05***. (C)** Representative appearance of plant height of null14, aflM14, null16 and aflM16 in T3 generation at 50 days after planting. **(D)** Representative appearance of dehusked mature maize ears from null14, aflM14, null16, and aflM16 at harvest (T4).

T1 to T2 generation plants from each of the four transgenic events were evaluated for height and kernel number per ear. Five to eleven plants per event were measured for height at the silk stage. Three to ten ears per event were counted to determine seed number. Plant height (T1) and number of T2 kernels per ear were not significantly different between transgenic and non-transgenic plants and also among events (Figures 3A,B). The T3 generation transgenic plants and the resulting mature ears (T4) harvested from these plants of aflM14 and aflM16 events also showed no phenotypic differences compared to null14 and null16 (Figures 3C,D).

### AFB_1_ Production in T3 and T4 Generation Homozygous Seeds

**FIGURE 4:**
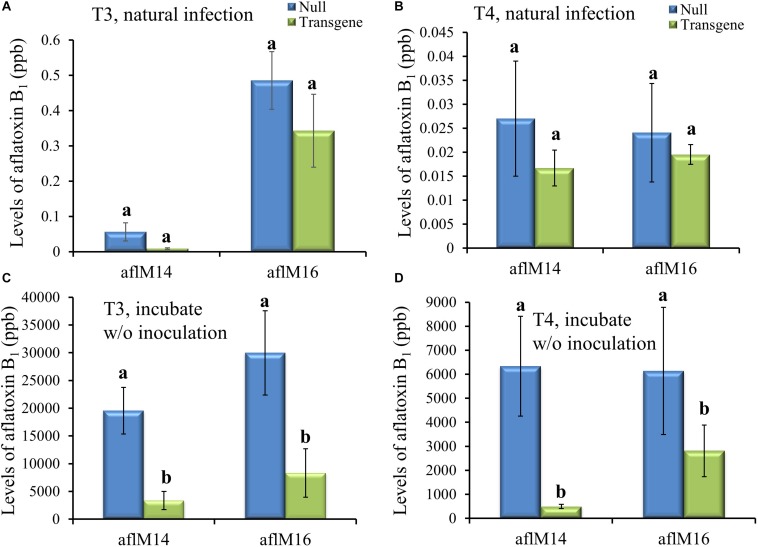
Aflatoxin production in the transgenic and null kernels of two different events in 2016 and 2017. Aflatoxin production in T3 (2016) generation **(A, C)** and T4 (2017) generation **(B, D)** transgenic and null kernels without *aflM* under field natural infection **(A, B)** and after incubation under laboratory Kernel Screening Assay conditions without inoculation **(C, D)**. Data are the mean and standard errors of 12–36 replicates of each event. Bars with different letters are significantly different at *P* ≤ 0.05. Transgene represents the kernels that contain *aflM* gene. Null represents the segregating non-transgenic kernels from the same event.

Mature kernels from non-inoculated ears grown in the field in 2016 (T3) and in 2017 (T4) were ground and analyzed for aflatoxin levels in these kernels under natural infection. Only very low levels (<0.6 ppb for aflM16 in 2016 and <0.04 ppb for both lines in 2017) of aflatoxin was detected in those kernels, and there was no difference between transgenic lines and the null controls (Figures 4A,B). However, significantly high levels of aflatoxin were detected after these kernels were surface-sterilized and incubated under 100% humidity at 30°C for 7 days without inoculation (Figures 4C,D), indicating the presence of sufficient levels of *A. flavus* inoculum inside the naturally infected kernels. It is also clear that the transgenic kernels had significantly lower levels of aflatoxin than their null controls for both events from both years (*P* ≤ 0.003–0.05), with 54.2–95.3% reduction in aflatoxin production compared to that in null kernels (Figures 4C,D).

**FIGURE 5:**
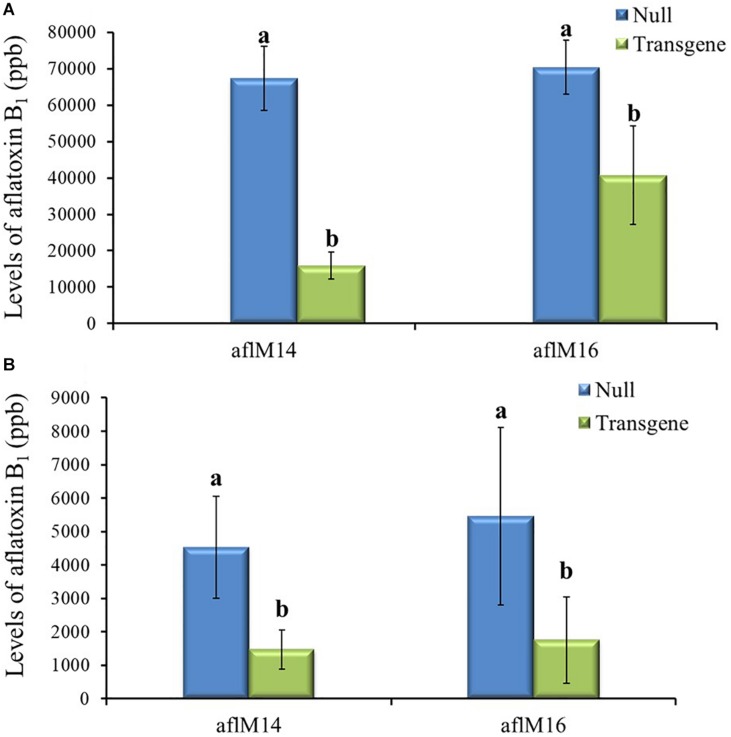
Aflatoxin production in the transgenic and null kernels of two different events in 2016 and 2017 under field inoculation conditions. Aflatoxin production in T3 (2016) generation **(A)** and T4 (2017) generation **(B)** transgenic and null kernels without *aflM* under field inoculation condition. Data are the mean and standard errors of 28–36 replicates of each event. Bars with different letters are significantly different at *P* ≤ 0.05. Transgene represents the kernels that contain *aflM* gene. Null represents the segregating non-transgenic kernels from the same event.

Mature kernels from field inoculated T3 and T4 generation ears were also analyzed for aflatoxin levels. The field-inoculated T3 generation kernels homozygous for *aflM* showed significantly reduced (up to 42.2–76.4%) aflatoxin contamination compared to kernels from the null (segregating non-transgenic) (*P* ≤ 0.04) for both aflM14 and aflM16 events (Figure 5A). Homozygous transgenic kernels from T4 generation also contained significantly less (68.0% reduction) aflatoxin than the null control under field inoculation conditions in 2017 (*P* ≤ 0.04) (Figure 5B). Overall, significant reduction in aflatoxin production was observed for transgenic maize lines in field inoculation and in incubation of naturally infected kernels under KSA conditions. These results demonstrated clearly that HIGS targeting of the *aflM* gene significantly reduced aflatoxin production in the homozygous transgenic kernels.

### Zygosity and AflM Transgene Copy Number Estimation in Different Transformation Events

In order to identify homozygous T2 seedlings from the segregating T2 population for seed increase, genomic DNA from T2 seedlings and T0 leaf tissues of the two events was used to determine the threshold number of cycles of *adh1* reference gene and the target *aflM* gene in each of the samples, which were then used to estimate zygosity of the T2 plants based on the ratio of aflM copy number in T2 vs in T0: 2^[(T2 Ct^*^(adh*1*)–^*^T2Ct (^*^*aflM*^*^)]–[T0 Ct^ *^(adh*1*^*^)–T0 Ct(^*^*aflM*^*^)]^ (Table 1). The ratio for homozygous seedlings from AflM14 event ranged from 1.90 to 2.03 and for the AflM16 event was from 6.40 to 6.55. This high value indicated the possible presence of multi-copies of target gene in the AflM16 event. However, our number of transgene integrations based on target gene segregation in T2 seedlings and Chi-square analysis indicated that both events have a single integration (Supplementary Table 3). To resolve this apparent conflicting information, the more accurate, Southern blot hybridization equivalent, droplet digital PCR was also performed to verify the copy number of the above transgenic lines using genomic DNA from T0 and T4 seedlings. The ratio of calculated gene copy number of *bar*/*adh1* for genomic DNA samples from aflM14(T0), aflM16(T0), aflM17(T0) ranged from 0.5 to 0.54 (Table 2), confirming these three events are single-copy hemizygous for the transgene. The droplet digital PCR also confirmed that aflM14(T4) and aflM16 (T4) are homozygous for the transgene based on the ratio of calculated gene copy number of *bar*/*adh1* (ranging from 0.86 to 0.96) (Table 2).

**TABLE 1:** Zygosity estimation of T2 seedling population from both aflM14 and aflM16 events using real time PCR.

Independent transgenic plant	2^(T2 Ct^*^(adh)–^*^T2Ct (^*^*aflM*^*^)^/2^(T0 Ct^ *^(adh*1*^*^)–T0 Ct(^*^*aflM*^*^)^		
	
	Heterozygous	Homozygous	Ratio
aflM14-1	0.87 ± 0.14	1.90 ± 0.11	1:2.2 ± 0.22
aflM14-2	1.05 ± 0.07	2.03 ± 0.05	1:1.9 ± 0.14
aflM16-1	3.09 ± 0.02	6.55 ± 0.46	1:2.1 ± 0.13
aflM16-2	3.12 ± 0.01	6.40 ± 0.04	1:2.04 ± 0.02

**TABLE 2:** Transgene copy number analysis through droplet digital PCR of genomic DNA from leaf tissues of T0 and T4 transgenic plants.

Event	Bar copy/20 μL	Adh1 copy/20 μL	Bar/Adh1	Copy number
aflM14 (T0)	278	514	0.54	1 (hemi)
aflM16 (T0)	414	828	0.5	1 (hemi)
aflM17 (T0)	342	660	0.52	1 (hemi)
aflM14 (T4)	1110	1154	0.96	1 (homo)
aflM16 (T4)	666	774	0.86	1 (homo)

*The target gene copy number was calculated based on the ratio of number of target *bar* gene (phosphinothricin acetyltransferase gene) molecules in the construct compared to maize single copy reference alcohol dehydrogenase gene (*adh1*) in the genomic DNA samples.*

### Crossing of the Transgene Into Elite Inbred Lines Resulted in Reduced AFB_1_ Production in the F1 Crosses

**FIGURE 6:**
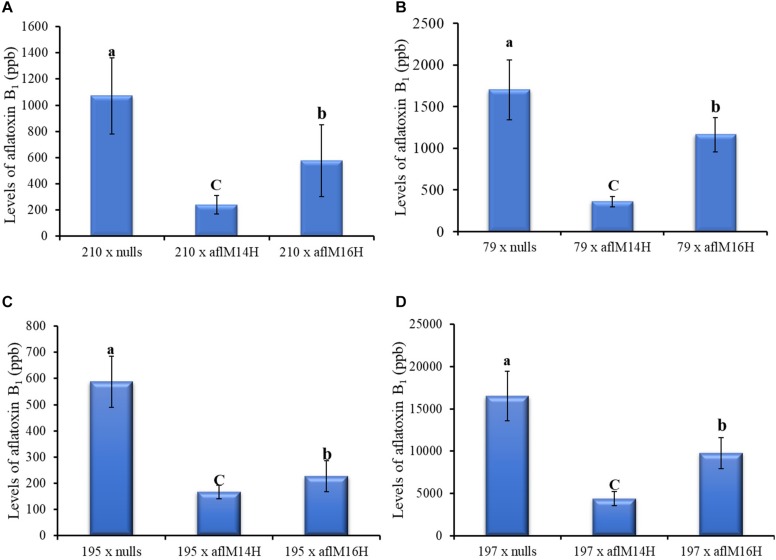
Aflatoxin production in crosses of two non-stiff stalk (**A**, PHW79 or 79; and **B**, LH210 or 210) and two stiff stalk (**C**, LH195 or 195; and **D**, LH197 or 197) elite inbred lines with aflM14 (homozygous and null) or aflM16 (homozygous and null) lines under field inoculation conditions. At least eight ears per treatment were collected. Kernels from each half ear were ground and three subsamples were analyzed for aflatoxin levels using HPLC. The aflatoxin data of elite line crossing with nulls were a combined data from elite line crossing with both aflM14N and aflM16N. Data presented here are the mean and standard errors of at least 24 replicates of each cross. Bars with the same letter were not significantly different at *P* ≤ 0.05. All of the analyses were done using non-transformed raw data except those from PHW79 × aflM16H and PHW79 × aflM16N, which were log transformed to reduce sample variation.

In crosses with non-stiff stock elite inbred lines, the resulting kernels of LH210 × aflM14H (homo) or aflM16H produced significantly less aflatoxin (60–80% reduction) compared to those in the kernels of LH210 × aflM14N (null) or aflM16N with *P* = 0.0056 and *P* = 0.0452, respectively, under field inoculation conditions (Figure 6A). The kernels of PHW79 × aflM14H (homo) or aflM16H crosses also supported significantly less aflatoxin compared to those in the kernels of PHW79 × aflM14N (null) or aflM16N with *P* = 0.0023 and *P* = 0.02, respectively (Figure 6B). In crosses with stiff stock lines (LH195 and LH197) and under field inoculation conditions, the resulting kernels of LH195 and LH197 × aflM14H or aflM16H crosses supported significantly less aflatoxins compared to those in the kernels of LH195 and LH197 × aflM14N (null) or aflM16N crosses with *P* values ranging from 0.0001 to 0.0183 (Figures 6C,D). In addition, among the four inbred lines used in the crosses, LH197 appeared to be the most susceptible one and supported 10 times more aflatoxin production than PHW79, 15 times more than LH210, and 30 times more than LH195, which is the most resistant one (Figure 6).

### Detection of High Levels Gene-Specific Small RNAs in the Transgenic Leaf and Kernel Tissues

In order to determine whether the enhanced aflatoxin resistance in the homozygous transgenic kernels compared to the null was due to the presence of *aflM* specific small RNA produced from the introduced RNAi vector, small RNAs from T0 and T3 leaf tissues and from T4 kernel tissues were sequenced and analyzed. The total number of reads from the libraries of aflM14 homo and aflM14 null T3 leaf tissues was about 40 and 30 million (Table 3), respectively, and the total number of reads for B104 was over 62 million. After filtering out the reads that were aligned to the maize genome, 3,164 reads from the leaf tissue of aflM14 homo transgenic plants were specifically aligned to the *aflM* target gene, whereas only 4 and 1 reads from the aflM14 null and B104 controls were aligned to the *aflM* gene (Table 3), respectively. The total number of small RNA reads derived from the immature kernel tissues of T4 generation aflM14 and aflM16 were 1,532,829 and 1,800,225 (Table 3), respectively. Three hundred fifty-nine and 197 reads were *aflM* specific for the aflM14 and aflM16 events, respectively (Table 3), compared to 1 and 2 *aflM*-specific reads observed for the null aflM14 and null aflM16 controls (Table 3). These results are consistent with data obtained from T0 leaf tissue of alM14 and aflM16 that were collected in 2013 from greenhouse grown plants (Table 3).

**TABLE 3:** Number of small RNA reads in leaf tissues and immature kernel tissues of transgenic and non-transgenic maize lines.

Tissue type*	**Events**	**Total read**	**Reads aligned to maize genome**	**Reads aligned to *A. flavus* > 1 times**	**Reads aligned to *A. flavus* 1 time**	**Reads aligned to *aflM***
Leaf tissue (T0) collected in 2013	aflM14	1,300,823	834,079	107	2,076	1,372
	aflM16	1,254,164	963,063	74	1,706	1,256
	aflM11 (null)	1,203,478	869,307	60	1,301	3

Leaf tissue (T3) collected in 2016	aflM14 homo	40,222,099	30,003,837	1,233	5,894	3,164
	aflM14 null	30,795,339	29,030,160	17,516	26,750	4
	B104 (WT)	62,902,688	61,179,007	5,285	6,236	1

Immature kernels (T4) collected in 2017	aflM14 homo	1,532,829	989,008	86	670	359
	aflM14 null	1,552,692	1,111,600	446	86	1
	B104 (WT)	1,367,547	796,105	670	732	0
	aflM16 homo	1,800,225	1,163,932	88	599	197
	aflM16 null	1,588,892	1,294,348	45	321	2

* The small RNA libraries from T3 leaf tissues collected in 2016 were sequenced on Illumina HiSeq 2500 Platform at NC State University and the small RNA libraries from T0 leaf tissues collected in 2013 and T4 immature kernel tissues collected in 2017 were sequenced on Illumina HiSeq 4000 Platform in 2017 at UC Davis.

**FIGURE 7:**
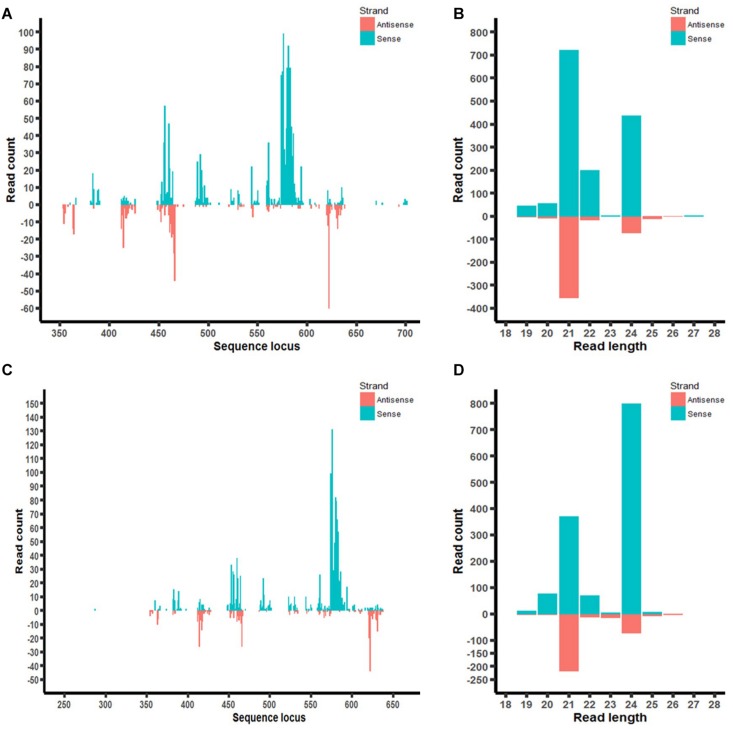
Small RNA profiling (RNAseq analysis) of *aflM* target gene in transgenic maize leaf tissue. **(A,C)**: Distribution of *aflM* specific small RNAs isolated from T0 leave tissue of aflM14 **(A)** and aflM16 **(C)** aligned to the target gene sequence. **(B,D)**: Read length distribution of sRNAs mapped to *aflM* from leaf tissue of aflM14 **(B)** and aflM16 **(D)**.

Furthermore, the distribution of *aflM*-specific small RNAs on the target gene was also examined in aflM14 and aflM16 (Figures 7A,C). Based on the small RNA distribution map, most of the small RNA appeared to be generated from a few hot spots in the 330 bp target sequence. The results of the small RNA distribution in the target gene were similar for both events (Figures 7A,C). However, the most abundant small RNA in aflM14 was 21 nt in length, followed by one that was 24 nt long (Figure 7B), whereas in aflM16, the most abundant small RNA was 24 nt in length, followed by one that was 21 nt long (Figure 7D).

## Discussion

The present study investigated the changes in aflatoxin resistance in transgenic maize lines containing HIGS construct targeting *aflM* of *A. flavus* in two independent events and found that both homozygous transgenic lines produced significantly less aflatoxins under repeated field inoculation studies. This enhanced aflatoxin resistance in the transgenic lines coincides with the presence of high levels of gene- specific small RNAs in their leaf and kernel tissues. Transferring this gene into elite inbred lines through crossing also led to enhanced aflatoxin resistance in the resulting F1 crosses containing the transgene. This study demonstrates that reduction of aflatoxin production through HIGS targeting the *A. flavus* aflatoxin biosynthesis pathway genes can be a practical and sustainable approach to manage aflatoxin contamination in maize and other susceptible crops.

During initial evaluation of different independent transformation events and later characterization of the progenies of aflM14 and aflM16 events, it was clear that transgenic lines developed from different events had different efficacy in reducing aflatoxin production. Homozygous transgenic kernels from the aflM14 event always produced less aflatoxin than those from the aflM16 event. One possible reason could be the dosage (copy number) effect. Therefore, real time PCR was first attempted to determine the target *aflM* gene copy number using the single copy *adh1* gene as a reference, which suggested that aflM16 could have multiple copies of the target gene (Table 1). However, due to the well-known varying accuracy (ranging from 14 to 100%) of real time PCR in gene copy number assessment (Bubner and Baldwin, 2004) and the apparent contradiction to our number of transgene integration loci calculation based on chi-square analysis (Supplementary Table 3), the droplet digital PCR was performed using the same genomic DNA samples, which confirmed both events to have a single copy of integration. The accuracy of droplet digital PCR in comparison to Southern blot analysis in determining gene copy number has been well established (Głowacka et al., 2016; Collier et al., 2017). Another possible explanation of such differences is the result of random integration of the T-DNA into the maize genome during the initial *Agrobacterium* transformation process. The position of the T-DNA insertion in chromosome and the chromatin structure of the area surrounding the transgene insertion can influence transgene expression (Dean et al., 1988; Peach and Velten, 1991; Breyne et al., 1992). Such a “chromosomal position effect” has been widely reported (Alberts and Sternglanz, 1990; Kumpatla et al., 1998; Matzke and Matzke, 1998), even though not all event-to-event variation can be explained by such an effect, according to Petolino and Kumar (2016).

A 42–76% reduction under field condition and 54.2–95.3% reduction under KSA in aflatoxin production was observed in the two events compared to the controls, which is similar to what has been reported in earlier studies in transgenic maize and peanut using similar approaches (Masanga et al., 2015; Thakare et al., 2017; Sharma et al., 2018). However, our data were based on much larger sample sizes and on multi-year field studies with additional laboratory KSA confirmations as well as highly sensitive HPLC analysis of aflatoxin B_1_ production.

The intrinsically high variation of aflatoxin production among different maize kernels of the same line makes evaluating changes in aflatoxin resistance of the HIGS construct-containing transgenic maize lines a challenge. In order to reduce such variations under field inoculation conditions and get a true assessment of aflatoxin levels, four different sites per ear were inoculated with *A. flavus*, multiple kernels surrounding each inoculation site were collected and up to 15 ears per line were inoculated for analysis of toxin production. This field inoculation study was also conducted over a period of three years to further rule out any possible impact caused by environmental differences, which have been known to affect aflatoxin production in maize (Cotty et al., 2007; Fountain et al., 2014). In addition, KSA was performed to verify the levels of toxin production under more uniform inoculation and more controlled environmental conditions. The toxin data from both the field and KSAs showed good agreement among them. To the best of our knowledge, this is the first report demonstrating the efficacy of HIGS in reducing aflatoxin contamination through both repeated field and laboratory studies.

Analysis of naturally infected kernels only detected very low levels of aflatoxin in both transgenic and control kernels, indicating the necessity of performing artificial inoculations to separate the resistance between transgenic and control line. Our field inoculations and KSAs subjected the kernels to extremely high inoculum concentrations, under which the transgenic plants still had significantly less aflatoxin than the controls. The aflatoxin levels in the inoculated transgenic lines, however, were still much higher than the 20 ppb limit set by FDA (Park and Liang, 1993). These plants are unlikely to encounter such extremely high inoculum concentration under natural infection conditions. Therefore, it is reasonable to speculate that the toxin levels in these transgenic lines under natural infection conditions would be much lower than under artificial inoculation conditions. Field inoculation studies conducted in 2017 and 2018 also supported the above speculation. Overall aflatoxin production was much lower in both control and transgenic lines in 2018 when the inoculum concentration was reduced from 4 × 10^6^ in 2016 to 1 × 10^5^ conidia/mL.

Sequencing of small RNA libraries constructed from T0 and T3 leaf tissues as well as from T4 kernel tissues confirmed the presence of high levels of gene-specific small RNAs in the homozygous transgenic leaf and kernel tissues compared to B104 and null controls. These high levels of gene specific small RNAs can only come from the HIGS RNAi vector since all the samples used for small RNA sequencing study were from field-grown or greenhouse (T0 leaf) plants without inoculations, indicating the observed enhanced aflatoxin resistance in the transgenic lines was due to the siRNAs produced from the transformed HIGS vector. The RNA sequencing study also revealed that the double 35S promoter used in the present study drove more gene specific small RNAs expression in the leaf tissues than in the kernel tissues. Future studies should use a seed-specific and stress- or infection-inducible promoter to reduce the energy cost and possible yield reduction due to constant expression of the transgene in the whole transgenic plants.

Several recent studies have reported suppression of fungal diseases through direct applications of dsRNA (Koch et al., 2016; Wang and Jin, 2017; McLoughlin et al., 2018; Song et al., 2018). In addition, small RNAs have been reported to be transported locally from cell to cell through plasmodesmata and over long distances through plant phloem systems (Liu and Chen, 2018). Therefore, future studies could also examine the feasibility of direct application of *in vitro* synthesized dsRNA targeting *aflM* as a more practical and effective way of managing aflatoxin contamination in maize and other susceptible crops. Although this GMO-free RNAi approach is appealing (Dalakouras et al., 2019), one factor that may limit the direct application of dsRNA as a practical disease control approach is the lack of sufficient secondary amplification (Song et al., 2018). Successful disease control may require frequent reapplication of dsRNA to maintain a high level of dsRNA on leaf surface for this to work (Song et al., 2018). In comparison with external application of dsRNAs, genetic transformation to suppress the fungal target genes through HIGS is likely to result in more consistent presence of high levels of siRNA and to offer a more sustainable approach in managing aflatoxin contamination in maize and other susceptible crops.

## Data Availability Statement

The datasets generated for this study can be found in the NCBI SRA database under the following accession number: PRJNA577960.

## Author COntributions

YR and Z-YC designed the research. YR, OO, QW, and Z-QH performed the research. YR and DH analyzed the data. YR and Z-YC wrote the manuscript. KW directed the production of the maize transgenic lines; JC and KR provided technical support.

## Conflict of Interest

The authors declare that the research was conducted in the absence of any commercial or financial relationships that could be construed as a potential conflict of interest.

## Footnotes

1 www.fas.usda.gov/psdonline
