# Supplementary material for: Host Induced Gene Silencing Targeting Aspergillus flavus aflM Reduced Aflatoxin Contamination in Transgenic Maize Under Field Conditions
Source: Front Microbiol. 2020 Apr 28;11:754. doi: 10.3389/fmicb.2020.00754 (PMC7201132; doi:10.3389/fmicb.2020.00754)
Supplement: Supplementary file 1 [file Table_1.docx]

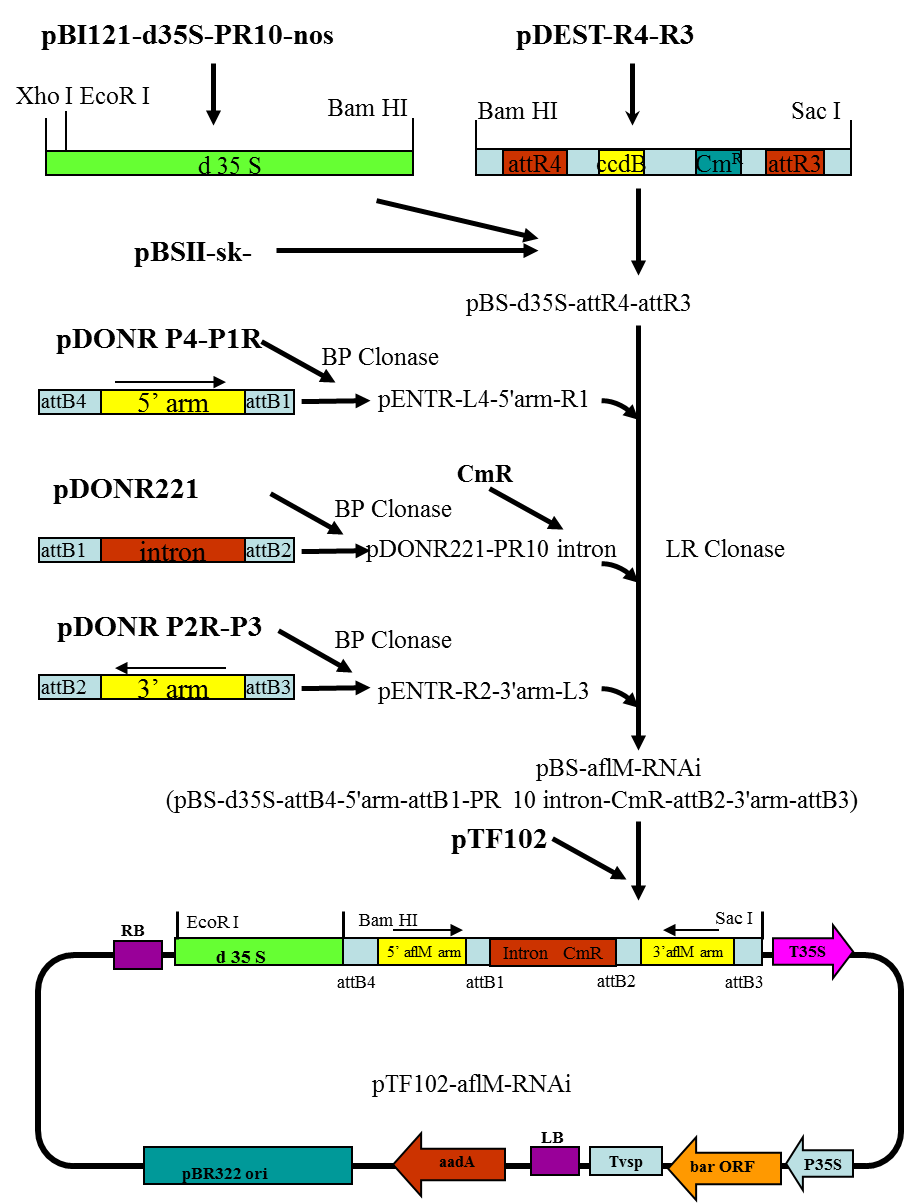


**Supplemental Figure 1:** Construction scheme of versicolorin (ver-1/aflM) gene silencing vector. The double 35S promoter and the DNA region containing the attR4-attR3 cassette were amplified by polymerase chain reaction (PCR) separately from their corresponding vectors, and cloned into the corresponding restriction sites in pBluesript II SK- vector. The DNA regions corresponding to the PR10 5′arm, intron and 3′ arm were amplified by PCR with primers containing unique homologous recombination sites cloned into their corresponding entry vectors. A chloramphenicol resistance gene (CmR) selection marker was then inserted into the middle of the PR10 intron before the LR clonase reaction to assemble the RNAi cassette into the pBluescript vector to produce the pBS-d35S-attB4-5′arm-attB1-PR10 intron-CmR-attB2-3′arm-attB3 vector (named pBS-PR10-RNAi). The RNAi cassette was then cloned into the pTF102 vector through ligation to produce the final pTF102-aflM-RNAi vector. This figure is modified from Chen et al (2010).


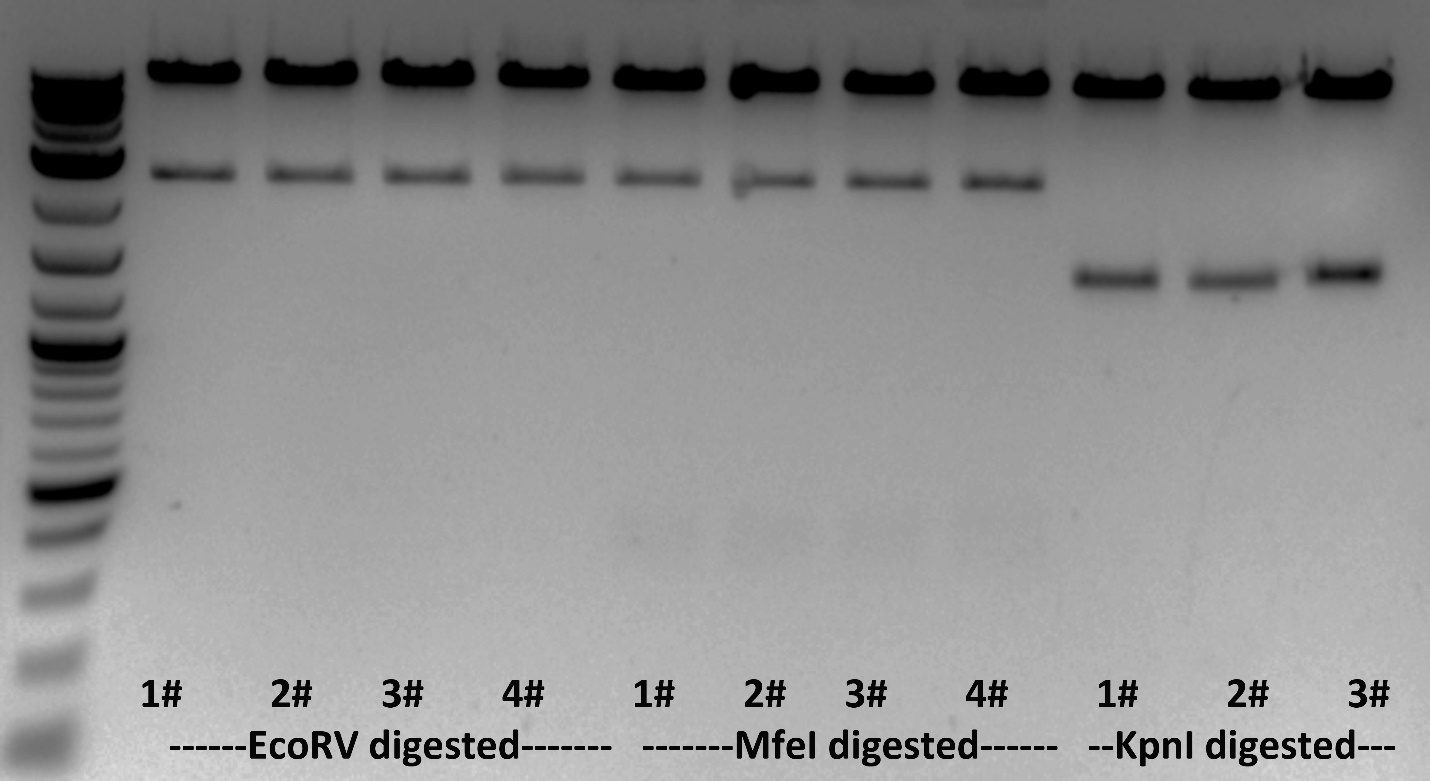


**Supplemental Figure 2:** Confirming the pTF102-aflM-RNAi construct by digested with *EcoR* V the expected sizes are 2447 and 9085 bp, *Mfe* I the expected sizes are 299, 2447, and 8786 bp, and *Kpn* I the expected sizes are 1328 and 10204 bp.

**Supplemental Table 1** List of primers used for constructing the Ti vector for suppressing the *aflM* and for zygosity and droplet digital PCR

| Primer name | Oligonucleotide sequence (5′→3′) |
| --- | --- |
| Ver-1-F | AACACCCGTGGCCAGTTC |
| Ver-1 R | ATGCCAGGAAGCTCACTACC |
| RT-Ver-F | CACCTTTGTTCGCTGCATG |
| RT-Ver-R | TGCTCATCGGTGAAAGTCTC |
| *Zm*18S-F | GAGAAACGGCTACCACATCCA |
| *Zm*18S-R | ACGCGCCCGGTATTGTTAT |
| attB4-Ver-F | *GGGGACAACTTTGTATAGAAAA*GTTGAACACCCGTGGCCAGTTC |
| attB1-Ver-R | *GGGGACTGCTTTTTTGTACAAACTTG*ATGCCAGGAAGCTCACTACC |
| attB2-Ver-F | *GGGGACAGCTTTCTTGTACAAAGTGG*TTCAAAGGCGAGAGCCAA |
| attB3-Ver-R | *GGGGACAACTTTGTATAATAAAGTTG* AACACCCGTGGCCAGTTC |
| d35S-F | ATGACGCACAATCCCACTATCCT |
| RNAi-R | GCCATACGGAATTCCGG |
| PR10-F | CACCTCAGTCATGCCGTTCA |
| RT-Ver-F (Taq) | GACTGCGGAGACAAGAAGAT |
| RT-Ver-R (Taq) | CTACCTGCTCATCGGTGAAA |
| RT-Ver-probe* | FAM-TTTGGCTGT/ZEN/GTCGCGGGAGTATAT-IBFQ |
| RT-Adh1-F (Taq) | CGTCGTTTCCCATCTCTTCCT CC |
| RT-Adh1-R (Taq) | CCACTCCGAGACCCTCAGTC |
| Adh1-probe* | HEX-AATCAGGGC/ZEN/TCATTTTCTCGCTCCTCA-IBFQ |
| RT-Bar-F | GGAAGTTGACCG TGCTTGT |
| RT-Bar-R | GATCTACCATGAGCCCAGAAC |
| Bar-probe* | FAM-CGATGTAGT/ZEN/GGTTGACGATGGTGCA-IBFQ |

Italics letters indicate the homologous recombinant site that attached to the end of the gene specific primer sequences. *: the probes were labeled with FAM (6-fluorescein) or HEX (hexachloro fluorescein) at the 5′end and double quenched with ZEN and Iowa Black FQ (IBFQ).

**Supplemental** **Table 2** Variations in T1 kernel number and average kernel weight among the 23 transgenic lines in seven transgenic events of HIGS-*aflM* in B104 that were produced at Iowa State University.

| Event | Line name | Number of kernels per ear | Kernel weight (g) *  (average) |
| --- | --- | --- | --- |
| AflM-9 | aflM-9-1 | 13 | 0.2226 |
| AflM-10 | aflM-10-1 | 103 | 0.1747 |
|  | aflM-10-3 | 70 | 0.1913 |
|  | aflM-10-4 | 57 | 0.1508 |
|  | aflM-10-5 | 33 | 0.1847 |
| AflM-11 | aflM-11-1 | 33 | 0.1968 |
|  | aflM-11-3 | 16 | 0.1809 |
| AflM-13 | aflM-13-1 | 59 | 0.1999 |
|  | aflM-13-2 | 20 | 0.2197 |
|  | aflM-13-3 | 83 | 0.2011 |
|  | aflM-13-4 | 72 | 0.1846 |
|  | aflM-13-6 | 145 | 0.1807 |
| AflM-14 | aflM-14-3 | 53 | 0.1980 |
|  | aflM-14-4 | 25 | 0.2238 |
|  | aflM-14-5 | 13 | 0.2150 |
|  | aflM-14-6 | 47 | 0.1637 |
| AflM-16 | aflM-16-2 | 76 | 0.2091 |
|  | aflM-16-4 | 53 | 0.2150 |
| AflM-17 | aflM17-2 | 36 | 0.2134 |
|  | aflM17-3 | 65 | 0.1945 |
|  | aflM17-4 | 68 | 0.1998 |
|  | aflM17-5 | 98 | 0.2005 |
|  | aflM17-6 | 68 | 0.19056 |

**Supplemental Table 3.** Number of transgene loci estimated based on genotyping of seedlings developed from self-pollinated T3 generation ears and chi-square analysis

| Event | AflM14 | | |  | AflM16 | | |
| --- | --- | --- | --- | --- | --- | --- | --- |
|  | Observed | Expected  (1 locus) | Expected  (2 loci) |  | Observed | Expected  (1 locus) | Expected  (2 loci) |
| Seedlings with transgene | 66 | 69 | 86.25 |  | 68 | 74.25 | 92.81 |
| Seedlings without transgene | 26 | 23 | 5.7 |  | 31 | 24.75 | 6.1 |
| Total number | 92 | 92 | 92 |  | 99 | 99 | 99 |
| X^2^ |  | 0.52 | 77.05 |  |  | 2.49 | 109.38 |
| P |  | 0.4701 | <0.0001 |  |  | 0.1142 | <0.0001 |
| Estimated # of integration | 1 | | |  | 1 | | |

The number of transgene integrations in the genome was estimated based on probability of calculated chi-square Χ^2^ = ∑ (observed-expected)^2^ / (expected) exceeding the critical value to reject or accept the null hypothesis of being one or two integrations. Segregation of seedlings with transgene and without (null) is expected to be 3:1 (transgene: null) for single integration or 15:1 for two integrations.
